# (3a*S*,9b*R*)-Methyl 1-methyl-3-phenyl-1,2,3,3a,4,9b-hexa­hydro­chromeno[4,3-*b*]pyrrole-3a-carboxyl­ate

**DOI:** 10.1107/S1600536808005199

**Published:** 2008-03-05

**Authors:** S. Nirmala, E. Theboral Sugi Kamala, L. Sudha, E. Ramesh, R. Raghunathan

**Affiliations:** a Department of Physics, Easwari Engineering College, Ramapuram, Chennai 600 089, India; b Department of Physics, SRM University, Ramapuram Campus, Chennai 600 089, India; c Department of Organic Chemistry, University of Madras, Guindy Campus, Chennai 600 025, India

## Abstract

In the title compound, C_20_H_21_NO_3_, the heterocyclic six-membered ring adopts a half-chair conformation and the pyrrolidine ring adopts an envelope conformation. The mol­ecular conformation is stabilized by C—H⋯O and C—H⋯N inter­actions.

## Related literature

For related literature, see: Brockmann & Tour (1995 ▶); Caine (1993 ▶); Carlson (1993 ▶); Cremer & Pople (1975 ▶); Di Natale *et al.* (1998 ▶); Nirmala *et al.* (2008 ▶); Sobral & Rocha Gonsalves (2001*a* ▶,*b* ▶); Sokoloff *et al.* (1990 ▶); Suslick *et al.* (1992 ▶); Tidey (1992 ▶); Wilner (1985 ▶).
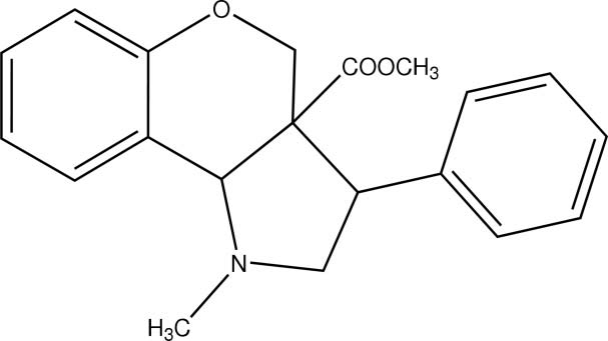


## Experimental

### 

#### Crystal data

C_20_H_21_NO_3_
*M* *_r_* = 323.38
Triclinic, 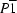

*a* = 7.9557 (4) Å
*b* = 10.2575 (7) Å
*c* = 10.2993 (8) Å
α = 79.626 (5)°
β = 87.361 (5)°
γ = 88.744 (4)°
*V* = 825.79 (9) Å^3^
*Z* = 2
Mo *K*α radiation
μ = 0.09 mm^−1^
*T* = 293 (2) K
0.20 × 0.20 × 0.12 mm

#### Data collection

Bruker Kappa APEX2 diffractometer
Absorption correction: multi-scan (Blessing, 1995 ▶) *T*_min_ = 0.983, *T*_max_ = 0.990
18704 measured reflections
4126 independent reflections
3051 reflections with *I* > 2σ(*I*)
*R*_int_ = 0.030

#### Refinement

*R*[*F* ^2^ > 2σ(*F* ^2^)] = 0.046
*wR*(*F* ^2^) = 0.143
*S* = 1.03
4126 reflections
218 parameters
H-atom parameters constrained
Δρ_max_ = 0.23 e Å^−3^
Δρ_min_ = −0.21 e Å^−3^

### 

Data collection: *APEX2* (Bruker, 2004 ▶); cell refinement: *APEX2*/*SAINT* (Bruker, 2004 ▶); data reduction: *SAINT*/*XPREP* (Bruker, 2004 ▶); program(s) used to solve structure: *SHELXS97* (Sheldrick, 2008 ▶); program(s) used to refine structure: *SHELXL97* (Sheldrick, 2008 ▶); molecular graphics: *ORTEP-3* (Farrugia, 1997 ▶); software used to prepare material for publication: *PLATON* (Spek, 2003 ▶).

## Supplementary Material

Crystal structure: contains datablocks I, global. DOI: 10.1107/S1600536808005199/bt2680sup1.cif

Structure factors: contains datablocks I. DOI: 10.1107/S1600536808005199/bt2680Isup2.hkl

Additional supplementary materials:  crystallographic information; 3D view; checkCIF report

## Figures and Tables

**Table 1:** Hydrogen-bond geometry (Å, °)

*D*—H⋯*A*	*D*—H	H⋯*A*	*D*⋯*A*	*D*—H⋯*A*
C5—H5*B*⋯N1	0.97	2.55	2.887 (2)	100
C12—H12⋯O2	0.98	2.43	2.781 (2)	101

**Fig. 1.**
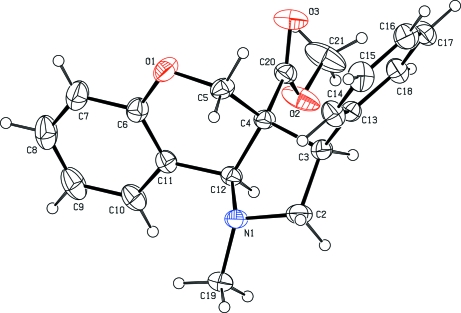
The molecular structure of the title compound with 30% probability displacement ellipsoids

**Fig. 2.**
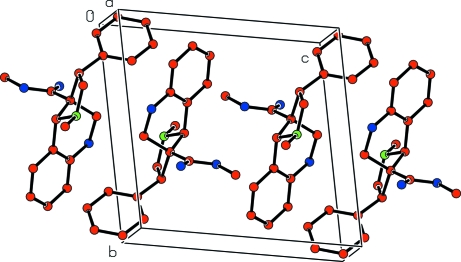
The packing of the molecules viewed down the a axis.

## supplementary crystallographic information

### Comment

Chromenopyrrole compounds are used in the treatment of impulsive disorders (Caine, 1993), aggressiveness (Tidey, 1992), parkinson's disease (Carlson, 1993), psychoses, memory disorders (Sokoloff *et al.*, 1990), anxiety and depression (Wilner, 1985). Pyrroles are also very useful precursors in porphyrin synthesis (Sobral & Rocha Gonsalves, 2001*a*, Sobral & Rocha Gonsalves, 2001*b*), and as monomers for polymer chemistry (Brockmann & Tour, 1995), with applications ranging from non – linear optical materials (Suslick *et al.*, 1992) to electronic noses (Di Natale *et al.*, 1998).

The bond lengths, bond angles and torsion angles of the title compound are comparable with the similar structure solved earlier (Nirmala *et al.*, 2008). The sum of bond angles around atom N1(331.7°) is in accordance with *sp*^3^ hybridization. The aromatic six-membered rings are oriented at an angle of 72.9 (5)° with respect to each other. The six-membered heterocyclic ring of the chromenopyrrole moiety adopts a half-chair conformation with puckering parameters of *q*_2_ = 0.357 (1) Å, *q*_3 _= -0.314 (1) Å and *φ*= -157.9 (2)° (Cremer and Pople, 1975). Atom C4 deviates by 0.631 Å from the least – square plane through the remaining five atoms. The pyrrolidine ring adopts an envelope conformation with puckering parameters of *q*_2_ = 0.438 (1) Å and *φ* = -39.8 (2)° (Cremer and Pople, 1975). Atom C12 deviates by 0.659 (4) Å from the least – square plane through the remaining four atoms.

No significant intermolecular π–π interactions are observed between two phenyl rings. Their centroids are separated by 3.9165 (1) Å. The molecular conformation is stabilized C— H···O and C— H···N interactions.

### Experimental

A solution of (*Z*)-methyl 2-((2 - formylphenoxy) methyl)-3- phenylacrylate (1 mmol), sarcosine (1 mmol), in anhydrous methanol (10 ml), was refluxed. Completion of the reaction was evidenced by TLC analysis. The solvent was removed in vacuum. The crude product was subjected to column chromatography on silica gel (100–200 mesh) using petroleum ether – ethyl acetate (7:3) as the eluent. Compound was recrystallized from methanol.

### Refinement

H atoms were placed in idealized positions and allowed to ride on their parent atoms, with C—H = 0.93, 0.98, 0.97 and 0.96 Å for aromatic, methine, methylene and methyl H respectively, and *U*_iso_(H) = 1.5*U*_eq_(C) for methyl H, and *U*_iso_(H) = 1.2*U*_eq_(C) for all other H atoms.

### Crystal data

**Table:**

C_20_H_21_NO_3_	*Z* = 2
*M**_r_* = 323.38	*F*_000_ = 344
Triclinic, *P*1	*D*_x_ = 1.301 Mg m^−^^3^
Hall symbol: -P 1	Mo *K*α radiation λ = 0.71073 Å
*a* = 7.9557 (4) Å	Cell parameters from 5117 reflections
*b* = 10.2575 (7) Å	θ = 2.6–28.1º
*c* = 10.2993 (8) Å	µ = 0.09 mm^−^^1^
α = 79.626 (5)º	*T* = 293 (2) K
β = 87.361 (5)º	Prism, colourless
γ = 88.744 (4)º	0.20 × 0.20 × 0.12 mm
*V* = 825.79 (9) Å^3^	

### Data collection

**Table:**

Bruker KappaAPEX2 diffractometer	4126 independent reflections
Radiation source: fine-focus sealed tube	3051 reflections with *I* > 2σ(*I*)
Monochromator: graphite	*R*_int_ = 0.030
*T* = 293(2) K	θ_max_ = 28.4º
ω and φ scans	θ_min_ = 2.6º
Absorption correction: multi-scan(Blessing, 1995)	*h* = −10→10
*T*_min_ = 0.983, *T*_max_ = 0.990	*k* = −13→13
18704 measured reflections	*l* = −13→13

### Refinement

**Table:**

Refinement on *F*^2^	Hydrogen site location: inferred from neighbouring sites
Least-squares matrix: full	H-atom parameters constrained
*R*[*F*^2^ > 2σ(*F*^2^)] = 0.046	*w* = 1/[σ^2^(*F*_o_^2^) + (0.0739*P*)^2^ + 0.1492*P*] where *P* = (*F*_o_^2^ + 2*F*_c_^2^)/3
*wR*(*F*^2^) = 0.143	(Δ/σ)_max_ < 0.001
*S* = 1.03	Δρ_max_ = 0.23 e Å^−^^3^
4126 reflections	Δρ_min_ = −0.21 e Å^−^^3^
218 parameters	Extinction correction: SHELXL97 (Sheldrick, 2008), Fc^*^=kFc[1+0.001xFc^2^λ^3^/sin(2θ)]^-1/4^
Primary atom site location: structure-invariant direct methods	Extinction coefficient: 0.024 (5)
Secondary atom site location: difference Fourier map	

### Special details

**Table:**

Geometry. All e.s.d.'s (except the e.s.d. in the dihedral angle between two l.s. planes) are estimated using the full covariance matrix. The cell e.s.d.'s are taken into account individually in the estimation of e.s.d.'s in distances, angles and torsion angles; correlations between e.s.d.'s in cell parameters are only used when they are defined by crystal symmetry. An approximate (isotropic) treatment of cell e.s.d.'s is used for estimating e.s.d.'s involving l.s. planes.
Refinement. Refinement of *F*^2^ against ALL reflections. The weighted *R*-factor *wR* and goodness of fit *S* are based on *F*^2^, conventional *R*-factors *R* are based on *F*, with *F* set to zero for negative *F*^2^. The threshold expression of *F*^2^ > σ(*F*^2^) is used only for calculating *R*-factors(gt) *etc*. and is not relevant to the choice of reflections for refinement. *R*-factors based on *F*^2^ are statistically about twice as large as those based on *F*, and *R*- factors based on ALL data will be even larger.

### Fractional atomic coordinates and isotropic or equivalent isotropic displacement parameters (Å^2^)

**Table:**

	*x*	*y*	*z*	*U*_iso_*/*U*_eq_	
C2	0.58609 (18)	0.33276 (15)	0.84198 (19)	0.0553 (4)	
H2A	0.6321	0.3161	0.9291	0.066*	
H2B	0.6651	0.2989	0.7811	0.066*	
C3	0.41543 (16)	0.26557 (14)	0.84521 (15)	0.0396 (3)	
H3	0.4205	0.2149	0.7730	0.047*	
C4	0.29028 (15)	0.38426 (13)	0.80439 (13)	0.0347 (3)	
C5	0.22346 (17)	0.44251 (15)	0.92274 (14)	0.0416 (3)	
H5A	0.1512	0.3786	0.9785	0.050*	
H5B	0.3170	0.4595	0.9742	0.050*	
C6	0.19789 (18)	0.65300 (15)	0.78280 (15)	0.0437 (3)	
C7	0.1233 (2)	0.77835 (17)	0.7635 (2)	0.0589 (5)	
H7	0.0357	0.7975	0.8203	0.071*	
C8	0.1789 (3)	0.87321 (18)	0.6611 (2)	0.0681 (5)	
H8	0.1299	0.9574	0.6494	0.082*	
C9	0.3069 (3)	0.84568 (17)	0.5748 (2)	0.0649 (5)	
H9	0.3438	0.9106	0.5049	0.078*	
C10	0.3803 (2)	0.72004 (15)	0.59337 (16)	0.0496 (4)	
H10	0.4655	0.7010	0.5345	0.060*	
C11	0.32870 (17)	0.62246 (13)	0.69811 (14)	0.0378 (3)	
C12	0.40618 (15)	0.48584 (13)	0.71947 (13)	0.0342 (3)	
H12	0.4335	0.4590	0.6342	0.041*	
C13	0.36182 (15)	0.17036 (13)	0.96874 (14)	0.0366 (3)	
C14	0.4024 (2)	0.18592 (16)	1.09382 (16)	0.0493 (4)	
H14	0.4679	0.2571	1.1041	0.059*	
C15	0.3472 (2)	0.09747 (18)	1.20362 (17)	0.0581 (4)	
H15	0.3762	0.1092	1.2872	0.070*	
C16	0.2497 (2)	−0.00770 (16)	1.19071 (18)	0.0549 (4)	
H16	0.2121	−0.0669	1.2652	0.066*	
C17	0.2083 (2)	−0.02486 (16)	1.06844 (19)	0.0545 (4)	
H17	0.1417	−0.0957	1.0590	0.065*	
C18	0.26509 (19)	0.06275 (14)	0.95821 (16)	0.0457 (4)	
H18	0.2376	0.0491	0.8749	0.055*	
C19	0.70301 (18)	0.53858 (16)	0.72905 (17)	0.0475 (4)	
H19A	0.6792	0.6314	0.7017	0.071*	
H19B	0.7314	0.4995	0.6528	0.071*	
H19C	0.7958	0.5274	0.7866	0.071*	
C20	0.14592 (16)	0.33900 (14)	0.73480 (15)	0.0402 (3)	
C21	0.0540 (3)	0.2860 (3)	0.5379 (2)	0.0892 (8)	
H21A	0.0932	0.2878	0.4480	0.134*	
H21B	−0.0460	0.3399	0.5394	0.134*	
H21C	0.0293	0.1965	0.5790	0.134*	
N1	0.55638 (13)	0.47446 (11)	0.79846 (12)	0.0380 (3)	
O1	0.13061 (13)	0.56333 (11)	0.88343 (11)	0.0525 (3)	
O2	0.18247 (17)	0.33696 (15)	0.60870 (12)	0.0735 (4)	
O3	0.01458 (14)	0.30253 (14)	0.78731 (13)	0.0663 (4)	

### Atomic displacement parameters (Å^2^)

**Table:**

	*U*^11^	*U*^22^	*U*^33^	*U*^12^	*U*^13^	*U*^23^
C2	0.0314 (7)	0.0475 (9)	0.0774 (12)	0.0001 (6)	0.0029 (7)	0.0128 (8)
C3	0.0345 (6)	0.0368 (7)	0.0449 (8)	0.0008 (5)	0.0034 (5)	−0.0021 (6)
C4	0.0303 (6)	0.0362 (7)	0.0352 (7)	−0.0009 (5)	0.0013 (5)	−0.0008 (5)
C5	0.0375 (7)	0.0475 (8)	0.0367 (7)	0.0044 (6)	0.0044 (5)	−0.0014 (6)
C6	0.0445 (7)	0.0421 (8)	0.0459 (8)	0.0049 (6)	−0.0105 (6)	−0.0102 (6)
C7	0.0607 (10)	0.0490 (9)	0.0705 (12)	0.0145 (8)	−0.0175 (8)	−0.0182 (8)
C8	0.0816 (13)	0.0407 (9)	0.0842 (14)	0.0108 (8)	−0.0357 (11)	−0.0102 (9)
C9	0.0842 (13)	0.0419 (9)	0.0646 (11)	−0.0123 (8)	−0.0285 (10)	0.0087 (8)
C10	0.0588 (9)	0.0441 (8)	0.0440 (8)	−0.0119 (7)	−0.0114 (7)	0.0008 (6)
C11	0.0410 (7)	0.0357 (7)	0.0369 (7)	−0.0036 (5)	−0.0096 (5)	−0.0045 (5)
C12	0.0335 (6)	0.0364 (7)	0.0319 (7)	−0.0044 (5)	0.0026 (5)	−0.0044 (5)
C13	0.0317 (6)	0.0327 (6)	0.0436 (8)	0.0012 (5)	−0.0033 (5)	−0.0023 (5)
C14	0.0550 (9)	0.0426 (8)	0.0505 (9)	−0.0078 (6)	−0.0114 (7)	−0.0055 (7)
C15	0.0734 (11)	0.0560 (10)	0.0427 (9)	0.0043 (8)	−0.0094 (8)	−0.0016 (7)
C16	0.0571 (9)	0.0445 (9)	0.0544 (10)	0.0041 (7)	0.0048 (7)	0.0118 (7)
C17	0.0491 (8)	0.0374 (8)	0.0730 (12)	−0.0097 (6)	−0.0068 (8)	0.0034 (7)
C18	0.0488 (8)	0.0376 (7)	0.0502 (9)	−0.0050 (6)	−0.0110 (6)	−0.0039 (6)
C19	0.0357 (7)	0.0492 (8)	0.0560 (9)	−0.0088 (6)	0.0068 (6)	−0.0059 (7)
C20	0.0356 (6)	0.0371 (7)	0.0452 (8)	−0.0037 (5)	−0.0020 (6)	0.0007 (6)
C21	0.0921 (15)	0.122 (2)	0.0567 (12)	−0.0647 (15)	−0.0106 (11)	−0.0160 (12)
N1	0.0288 (5)	0.0415 (6)	0.0416 (6)	−0.0025 (4)	0.0015 (4)	−0.0022 (5)
O1	0.0496 (6)	0.0525 (6)	0.0521 (7)	0.0144 (5)	0.0101 (5)	−0.0058 (5)
O2	0.0680 (8)	0.1103 (11)	0.0448 (7)	−0.0518 (8)	0.0016 (6)	−0.0162 (7)
O3	0.0368 (6)	0.0932 (10)	0.0680 (8)	−0.0181 (6)	0.0043 (5)	−0.0116 (7)

### Geometric parameters (Å, °)

**Table:**

C2—N1	1.4593 (19)	C11—C12	1.5020 (18)
C2—C3	1.532 (2)	C12—N1	1.4672 (17)
C2—H2A	0.9700	C12—H12	0.9800
C2—H2B	0.9700	C13—C14	1.380 (2)
C3—C13	1.5083 (19)	C13—C18	1.382 (2)
C3—C4	1.5657 (17)	C14—C15	1.377 (2)
C3—H3	0.9800	C14—H14	0.9300
C4—C20	1.5058 (19)	C15—C16	1.371 (3)
C4—C5	1.5211 (19)	C15—H15	0.9300
C4—C12	1.5298 (17)	C16—C17	1.358 (3)
C5—O1	1.4326 (16)	C16—H16	0.9300
C5—H5A	0.9700	C17—C18	1.380 (2)
C5—H5B	0.9700	C17—H17	0.9300
C6—O1	1.3542 (19)	C18—H18	0.9300
C6—C7	1.389 (2)	C19—N1	1.4464 (17)
C6—C11	1.394 (2)	C19—H19A	0.9600
C7—C8	1.364 (3)	C19—H19B	0.9600
C7—H7	0.9300	C19—H19C	0.9600
C8—C9	1.379 (3)	C20—O3	1.1901 (17)
C8—H8	0.9300	C20—O2	1.3215 (19)
C9—C10	1.388 (2)	C21—O2	1.442 (2)
C9—H9	0.9300	C21—H21A	0.9600
C10—C11	1.386 (2)	C21—H21B	0.9600
C10—H10	0.9300	C21—H21C	0.9600
			
N1—C2—C3	106.72 (11)	N1—C12—C4	101.59 (10)
N1—C2—H2A	110.4	C11—C12—C4	111.76 (10)
C3—C2—H2A	110.4	N1—C12—H12	110.0
N1—C2—H2B	110.4	C11—C12—H12	110.0
C3—C2—H2B	110.4	C4—C12—H12	110.0
H2A—C2—H2B	108.6	C14—C13—C18	117.52 (13)
C13—C3—C2	118.02 (13)	C14—C13—C3	123.24 (13)
C13—C3—C4	114.69 (10)	C18—C13—C3	119.23 (13)
C2—C3—C4	103.54 (11)	C15—C14—C13	120.87 (15)
C13—C3—H3	106.6	C15—C14—H14	119.6
C2—C3—H3	106.6	C13—C14—H14	119.6
C4—C3—H3	106.6	C16—C15—C14	120.52 (16)
C20—C4—C5	109.80 (11)	C16—C15—H15	119.7
C20—C4—C12	115.64 (11)	C14—C15—H15	119.7
C5—C4—C12	108.18 (11)	C17—C16—C15	119.55 (15)
C20—C4—C3	109.63 (11)	C17—C16—H16	120.2
C5—C4—C3	112.03 (11)	C15—C16—H16	120.2
C12—C4—C3	101.36 (10)	C16—C17—C18	120.03 (15)
O1—C5—C4	111.89 (11)	C16—C17—H17	120.0
O1—C5—H5A	109.2	C18—C17—H17	120.0
C4—C5—H5A	109.2	C17—C18—C13	121.50 (15)
O1—C5—H5B	109.2	C17—C18—H18	119.3
C4—C5—H5B	109.2	C13—C18—H18	119.3
H5A—C5—H5B	107.9	N1—C19—H19A	109.5
O1—C6—C7	115.99 (14)	N1—C19—H19B	109.5
O1—C6—C11	123.19 (12)	H19A—C19—H19B	109.5
C7—C6—C11	120.76 (15)	N1—C19—H19C	109.5
C8—C7—C6	119.85 (17)	H19A—C19—H19C	109.5
C8—C7—H7	120.1	H19B—C19—H19C	109.5
C6—C7—H7	120.1	O3—C20—O2	122.50 (14)
C7—C8—C9	120.78 (16)	O3—C20—C4	124.50 (14)
C7—C8—H8	119.6	O2—C20—C4	112.91 (11)
C9—C8—H8	119.6	O2—C21—H21A	109.5
C8—C9—C10	119.33 (17)	O2—C21—H21B	109.5
C8—C9—H9	120.3	H21A—C21—H21B	109.5
C10—C9—H9	120.3	O2—C21—H21C	109.5
C11—C10—C9	121.17 (17)	H21A—C21—H21C	109.5
C11—C10—H10	119.4	H21B—C21—H21C	109.5
C9—C10—H10	119.4	C19—N1—C2	111.63 (11)
C10—C11—C6	118.09 (14)	C19—N1—C12	114.08 (11)
C10—C11—C12	121.91 (13)	C2—N1—C12	105.98 (11)
C6—C11—C12	119.97 (12)	C6—O1—C5	117.33 (11)
N1—C12—C11	113.14 (11)	C20—O2—C21	115.98 (14)
			
N1—C2—C3—C13	−129.88 (13)	C3—C4—C12—C11	−164.30 (11)
N1—C2—C3—C4	−1.93 (17)	C2—C3—C13—C14	33.72 (19)
C13—C3—C4—C20	−79.76 (15)	C4—C3—C13—C14	−88.74 (16)
C2—C3—C4—C20	150.25 (13)	C2—C3—C13—C18	−147.30 (14)
C13—C3—C4—C5	42.41 (16)	C4—C3—C13—C18	90.25 (16)
C2—C3—C4—C5	−87.58 (14)	C18—C13—C14—C15	−0.4 (2)
C13—C3—C4—C12	157.53 (12)	C3—C13—C14—C15	178.62 (14)
C2—C3—C4—C12	27.54 (14)	C13—C14—C15—C16	−0.4 (3)
C20—C4—C5—O1	−67.14 (15)	C14—C15—C16—C17	0.4 (3)
C12—C4—C5—O1	59.90 (14)	C15—C16—C17—C18	0.4 (3)
C3—C4—C5—O1	170.79 (11)	C16—C17—C18—C13	−1.1 (2)
O1—C6—C7—C8	−177.44 (15)	C14—C13—C18—C17	1.1 (2)
C11—C6—C7—C8	−0.4 (2)	C3—C13—C18—C17	−177.91 (13)
C6—C7—C8—C9	1.0 (3)	C5—C4—C20—O3	−28.52 (19)
C7—C8—C9—C10	−0.4 (3)	C12—C4—C20—O3	−151.25 (15)
C8—C9—C10—C11	−0.9 (3)	C3—C4—C20—O3	94.97 (17)
C9—C10—C11—C6	1.6 (2)	C5—C4—C20—O2	154.86 (13)
C9—C10—C11—C12	179.79 (14)	C12—C4—C20—O2	32.13 (17)
O1—C6—C11—C10	175.95 (13)	C3—C4—C20—O2	−81.65 (15)
C7—C6—C11—C10	−0.9 (2)	C3—C2—N1—C19	−150.89 (14)
O1—C6—C11—C12	−2.3 (2)	C3—C2—N1—C12	−26.14 (16)
C7—C6—C11—C12	−179.18 (13)	C11—C12—N1—C19	−72.87 (15)
C10—C11—C12—N1	87.51 (16)	C4—C12—N1—C19	167.17 (11)
C6—C11—C12—N1	−94.28 (15)	C11—C12—N1—C2	163.91 (12)
C10—C11—C12—C4	−158.54 (13)	C4—C12—N1—C2	43.96 (13)
C6—C11—C12—C4	19.67 (18)	C7—C6—O1—C5	−168.00 (14)
C20—C4—C12—N1	−161.84 (11)	C11—C6—O1—C5	15.0 (2)
C5—C4—C12—N1	74.57 (12)	C4—C5—O1—C6	−44.61 (17)
C3—C4—C12—N1	−43.38 (12)	O3—C20—O2—C21	−0.8 (3)
C20—C4—C12—C11	77.23 (14)	C4—C20—O2—C21	175.94 (17)
C5—C4—C12—C11	−46.35 (14)		

### Hydrogen-bond geometry (Å, °)

**Table:**

*D*—H···*A*	*D*—H	H···*A*	*D*···*A*	*D*—H···*A*
C5—H5B···N1	0.97	2.55	2.887 (2)	100
C12—H12···O2	0.98	2.43	2.781 (2)	101
